# Long noncoding RNA *uc.230*/CUG-binding protein 1 axis sustains intestinal epithelial homeostasis and response to tissue injury

**DOI:** 10.1172/jci.insight.156612

**Published:** 2022-10-10

**Authors:** Ting-Xi Yu, Sudhakar Kalakonda, Xiangzheng Liu, Naomi Han, Hee K. Chung, Lan Xiao, Jaladanki N. Rao, Tong-Chuan He, Jean-Pierre Raufman, Jian-Ying Wang

**Affiliations:** 1 Cell Biology Group, Department of Surgery, University of Maryland School of Medicine, Baltimore, Maryland, USA.; 2 Baltimore Veterans Affairs Medical Center, Baltimore, Maryland, USA.; 3 Department of Surgery, The University of Chicago Medical Center, Chicago, Illinois, USA.; 4 Department of Medicine and; 5 Department of Pathology, University of Maryland School of Medicine, Baltimore, Maryland, USA.

**Keywords:** Gastroenterology, Inflammatory bowel disease, Surgery

## Abstract

Intestinal epithelial integrity is commonly disrupted in patients with critical disorders, but the exact underlying mechanisms are unclear. Long noncoding RNAs transcribed from ultraconserved regions (T-UCRs) control different cell functions and are involved in pathologies. Here, we investigated the role of T-UCRs in intestinal epithelial homeostasis and identified T-UCR *uc.230* as a major regulator of epithelial renewal, apoptosis, and barrier function. Compared with controls, intestinal mucosal tissues from patients with ulcerative colitis and from mice with colitis or fasted for 48 hours had increased levels of *uc.230*. Silencing *uc.230* inhibited the growth of intestinal epithelial cells (IECs) and organoids and caused epithelial barrier dysfunction. Silencing *uc.230* also increased IEC vulnerability to apoptosis, whereas increasing *uc.230* levels protected IECs against cell death. In mice with colitis, reduced *uc.230* levels enhanced mucosal inflammatory injury and delayed recovery. Mechanistic studies revealed that *uc.230* increased CUG-binding protein 1 (CUGBP1) by acting as a natural decoy RNA for miR-503, which interacts with *Cugbp1* mRNA and represses its translation. These findings indicate that *uc.230* sustains intestinal mucosal homeostasis by promoting epithelial renewal and barrier function and that it protects IECs against apoptosis by serving as a natural sponge for miR-503, thereby preserving CUGBP1 expression.

## Introduction

Besides its essential role in digestion and absorption, the mammalian intestinal epithelium is characterized by rapid and constant self-renewal and acts as a physical and biochemical barrier against a wide array of luminal noxious substances and the gut microbiome. The human small intestinal epithelium turns over every 4–5 days and this continuous, tightly regulated process of regeneration is accomplished by an active intestinal stem cell (ISC) population located at the crypt base (1, 2). ISCs divide daily, giving rise to proliferating progenitors that differentiate into absorptive or secretory lineages as they migrate toward the intestinal lumen along the crypt-villus axis (1–3). Apoptosis occurs both at the crypts, maintaining a balance between newly divided and surviving cells, and at the villous tips, where differentiated cells are lost (4). In response to stress, the maintenance of intestinal epithelial homeostasis entails the initiation of well-controlled mechanisms that require intestinal epithelial cells (IECs) to quickly alter their gene expression patterns to modulate proliferation, migration, differentiation, apoptosis, and cell-to-cell interactions (5–7). Nonetheless, intestinal epithelial integrity is commonly disrupted in a variety of diseases including inflammatory bowel disease (IBD) and critical surgical disorders with sepsis and shock. This may result in the translocation of luminal toxic substances and bacteria to the bloodstream, sometimes leading to multiorgan dysfunction and death (8, 9).

Substantial portions of the human genome (> 96%) are transcribed into a large array of noncoding RNAs (ncRNAs) such as long ncRNAs (lncRNAs), microRNAs (miRNAs), circular RNAs, endogenous small-interfering RNAs (siRNAs), and natural antisense transcripts (10, 11). LncRNAs are defined as transcripts spanning more than 200 nucleotides in length distinct from other well-characterized structural RNAs such as transfer and ribosomal RNAs. Unlike miRNAs, most lncRNAs are poorly conserved among species and dynamically expressed in tissue-, differentiation stage-, and cell type-dependent manners, and the levels of cellular lncRNAs are altered rapidly in response to pathophysiological stresses (12, 13). LncRNAs act as molecular scaffolds, decoys, or signals to control virtually every level of gene regulation, including chromatin remodeling, transcriptional and posttranscriptional processes, and protein metabolism (10–12). LncRNAs regulate a variety of cellular processes and are intimately involved in diverse human diseases by working jointly with miRNAs, RNA-binding proteins (RBPs), and occasionally other molecules (13–15). Emerging evidence indicates that several lncRNAs enriched in the gut epithelium, such as *H19*, *SPRY4-IT1*, *uc.173*, *circHIPK3*, and *Gata6*, are master regulators of intestinal epithelial homeostasis and participate in controlling mucosal renewal, adaptation, and repair after acute injury to restore gut mucosal barrier integrity and preserve host defenses (3, 9, 13–17).

Ultraconserved regions (UCRs) are elements that are absolutely conserved (100%) among the orthologous regions of the human, rat, and mouse genomes (18). RNAs transcribed from UCRs (T-UCRs) do not encode recognizable proteins and represent a class of novel endogenous lncRNAs implicated in many cell functions and human diseases (19–21). The expression patterns of T-UCRs change in response to biological stress and pathologies, and the levels of cellular *uc.349A*/*uc.352*, *uc.338*, *uc.8*, *uc.339*, *uc.63^+^*, and *uc.475* increase remarkably in cancer and/or after exposure to hypoxia (19, 22). *Uc.283^+^A* increases dramatically in cancer and is subject to epigenetic inactivation by CpG island hypermethylation (23). We recently reported that 21 T-UCRs, including *uc.230* and *uc.173*, are differentially expressed in the intestinal mucosa of fasted mice compared with nonfasted controls (16, 17, 24). Increasing *uc.173* levels enhance small intestinal mucosal growth by increasing the degradation of primary miR-195 (16). *uc.173* also promotes gut barrier integrity by stimulating the translation of the tight junctional (TJ) protein claudin-1 (CLDN1) and acting as a natural decoy to reduce the availability of miR-29b to *CLDN1* mRNA (17). To date, no studies have investigated the role of *uc.230* in regulating intestinal epithelial homeostasis.

Here, we provide evidence that expression levels of *uc.230* in intestinal mucosa are significantly altered in active ulcerative colitis (UC) and in mice with dextran sodium sulfate-induced (DSS-induced) colonic mucosal injury. Further, we show that *uc.230* is required for normal intestinal epithelial renewal and gut barrier function and protects IECs against apoptosis by acting as a natural sponge for miR-503, thereby preserving the expression of the RBP CUG-binding protein 1 (CUGBP1).

## Results

### Altered uc.230 levels in mouse and human intestinal mucosal injury.

**Figure 1:**
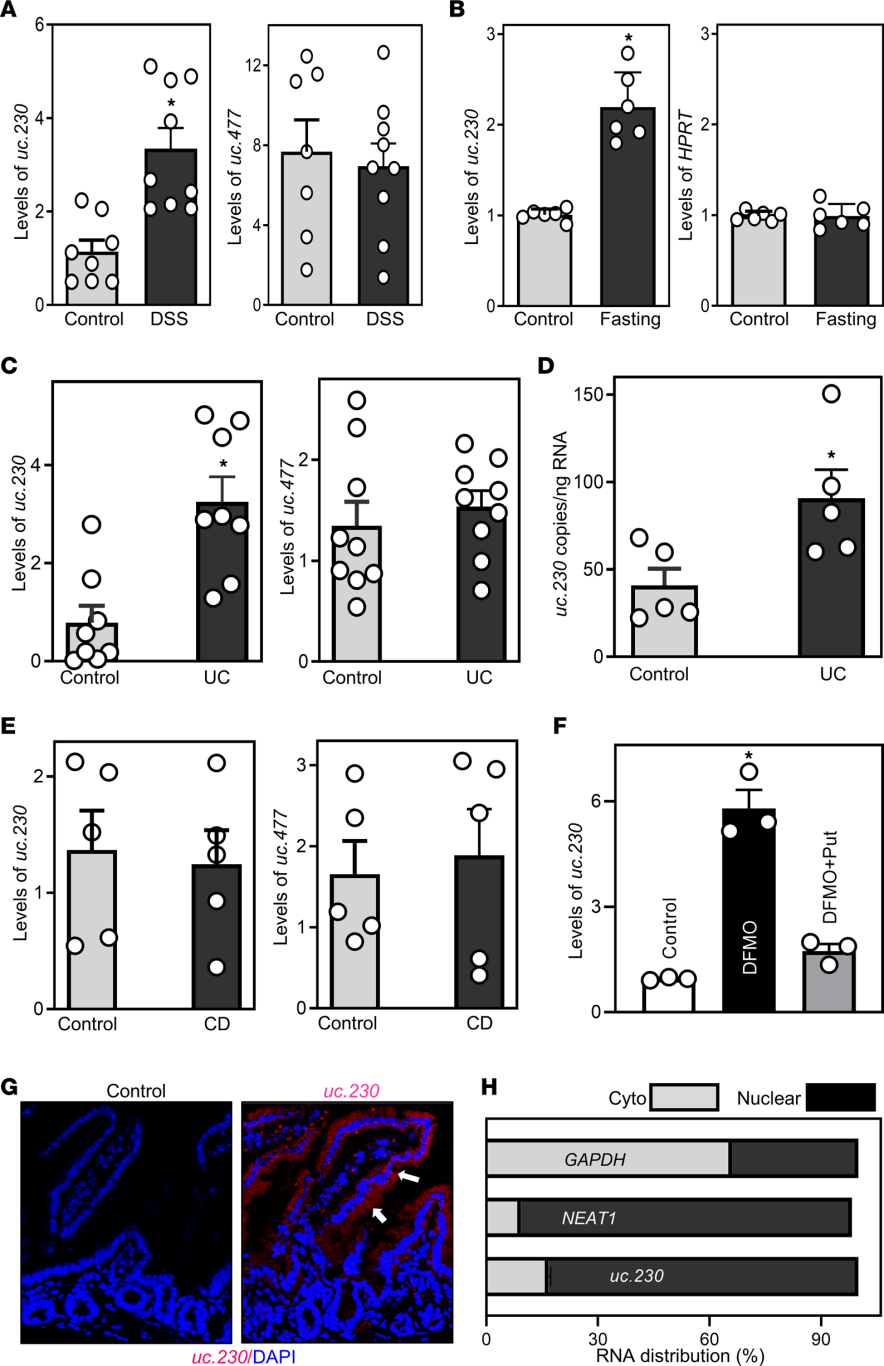
Mucosal *uc.230* expression associated with intestinal disease. (**A**) Levels of mucosal *uc.230* and *uc.477* in the colons of mice treated with water (control) or 3% DSS for 5 days were measured using qPCR. Values are the means ± SEM (*n* = 8 or 9). *P < 0.05 compared with control mice. (**B**) Levels of mucosal *uc.230* in the small intestines of mice fasted for 48 hours (*n* = 6). (**C** and **D**) Total levels and quantification of copy numbers of *uc.230* in the colonic mucosa of patients with active UC were measured by qPCR (*n* = 8) and digital qPCR analyses (*n* = 5), respectively. *P < 0.05 compared with controls. (**E**) Levels of tissue *uc.230* and *uc.477* in the ileal mucosa of patients with CD (*n* = 5). (**F**) Levels of *uc.230* in cultured Caco-2 cells exposed to DFMO (5 mmol/L) alone or plus putrescine (10 μmol/L) for 6 days. *P < 0.05 compared with control (*n* = 3). (**G**) Distribution of *uc.230* in the mucosa of the small intestine as measured by RNA-FISH. Total original magnification, 200×. (**H**) Levels of cyto and nuclear *uc.230*, lncRNA *NEAT1*, and *Gapdh* mRNA in Caco-2 cells. Statistical significance was analyzed using unpaired, 2-tailed Student’s *t* test except for results in **G** and **H**. All experiments in **G** and **H** were repeated 3 times with similar results. Put, putrescine; cyto, cytoplasmic.

We used murine models to explore the role of *uc.230* in maintaining intestinal epithelial homeostasis in response to stress; colonic mucosal injury and small intestinal mucosal atrophy were induced respectively using DSS and food starvation (25, 26). Consistent with our work (27) and others (25), after we administered 3% DSS in their drinking water for 5 days, mice developed colon epithelial damage, granulocyte infiltration, and bloody diarrhea, mimicking the injury observed in human ulcerative colitis. As shown in Figure 1A, mucosal *uc.230* abundance increased significantly in the colons of DSS-treated mice, achieving 3-fold higher levels than those observed in control animals. The specificity of this response was supported by the observation that DSS treatment did not alter colon mucosal levels of *uc.447*. As reported previously (16, 28), fasting mice for 48 hours inhibited small intestinal mucosal growth, confirmed by decreases in both the proliferating crypt cell population (marked by BrdU) and villus and crypt length. In fasted mice, inhibited growth of the small intestinal mucosa was also associated with a significant increase in tissue levels of *uc.230* without affecting the abundances of mucosal lncRNA *HPRT* (Figure 1B) and *uc.477*. The latter finding again supports the specificity of increases in *uc.230* in response to intestinal mucosal stress.

To examine the potential human disease relevance of these changes in *uc.230* abundance, we measured the levels of *uc.230* in human intestinal mucosal tissues. Colonic and ileal mucosal tissues were collected from patients with active UC and Crohn’s disease (CD), respectively, whereas tissue samples from subjects without gut mucosal injury, inflammation, or barrier dysfunction served as controls. As shown in Figure 1C, as determined using real-time quantitative PCR (qPCR) analysis, compared with control colonic tissues, in active UC, the colon mucosa exhibited significantly higher levels of *uc.230* but not *uc.477*. Digital qPCR analysis further showed that copy numbers of *uc.230* in the colonic mucosa were significantly greater in UC tissues compared with control (Figure 1D). For absolute quantification of the copy number, *uc.230* was read from 20,000 partitions from each tissue sample. In contrast to the findings in UC, compared with control ileal tissues, there were no significant differences in the levels of either *uc.230* or *uc.477* in ileal mucosal tissues obtained from CD subjects (Figure 1E).

Because polyamines are biological regulators of intestinal epithelial homeostasis (5, 29, 30), we examined the influence of decreasing the levels of cellular polyamines on *uc.230* expression. Studies conducted in cultured Caco-2 cells revealed that altered apoptosis and proliferation induced by polyamine depletion were also associated with changes in the levels of cellular *uc.230*. Consistent with our prior studies (29), treatment of cells for 6 days with D,L-α-difluoromethylornithine (DFMO), a specific inhibitor of polyamine biosynthesis (5), almost completely depleted the cellular polyamines putrescine and spermidine. Polyamine depletion by DFMO inhibited cell proliferation and reduced susceptibility to TNF-α/cycloheximide-induced (TNF-α/CHX-induced) apoptosis, as previously reported (29, 30). Interestingly, decreasing the levels of cellular polyamines with DFMO also increased *uc.230* abundance (Figure 1F), but not levels of *uc.477* or circRNA *circPABPN1*. A rescue experiment with exogenous putrescine given together with DFMO prevented the induction in cellular *uc.230* levels, associated with restored cell proliferation and susceptibility to TNFα/CHX-induced apoptosis. These findings support a role for polyamines in regulating cellular *uc.230* levels.

Notably, *uc.230* transcript sequences are conserved in human, mouse, and rat genomes (Supplemental Figure 1; supplemental material available online with this article; https://doi.org/10.1172/jci.insight.156612DS1). In murine small intestinal epithelium, *uc.230* was highly expressed in the villous area (Figure 1G), while levels were relatively low in the crypt region as measured by RNA-FISH. We also detected a predominant nuclear distribution of *uc.230* in Caco-2 cells (Figure 1H), like the subcellular localization of lncRNA *NEAT1*. On the other hand, *Gapdh* mRNA, a control, was detected in both the cytoplasm and nucleus. Taken together, these studies conducted in mice, human tissues, and cultured IECs indicate that expression levels of *uc.230* in the intestinal epithelium increase in response to various pathophysiological stresses, strongly supporting important roles for this lncRNA in maintaining intestinal epithelial integrity and the response to mucosal injury and growth inhibition.

### uc.230 is essential for IEC renewal.

**Figure 2:**
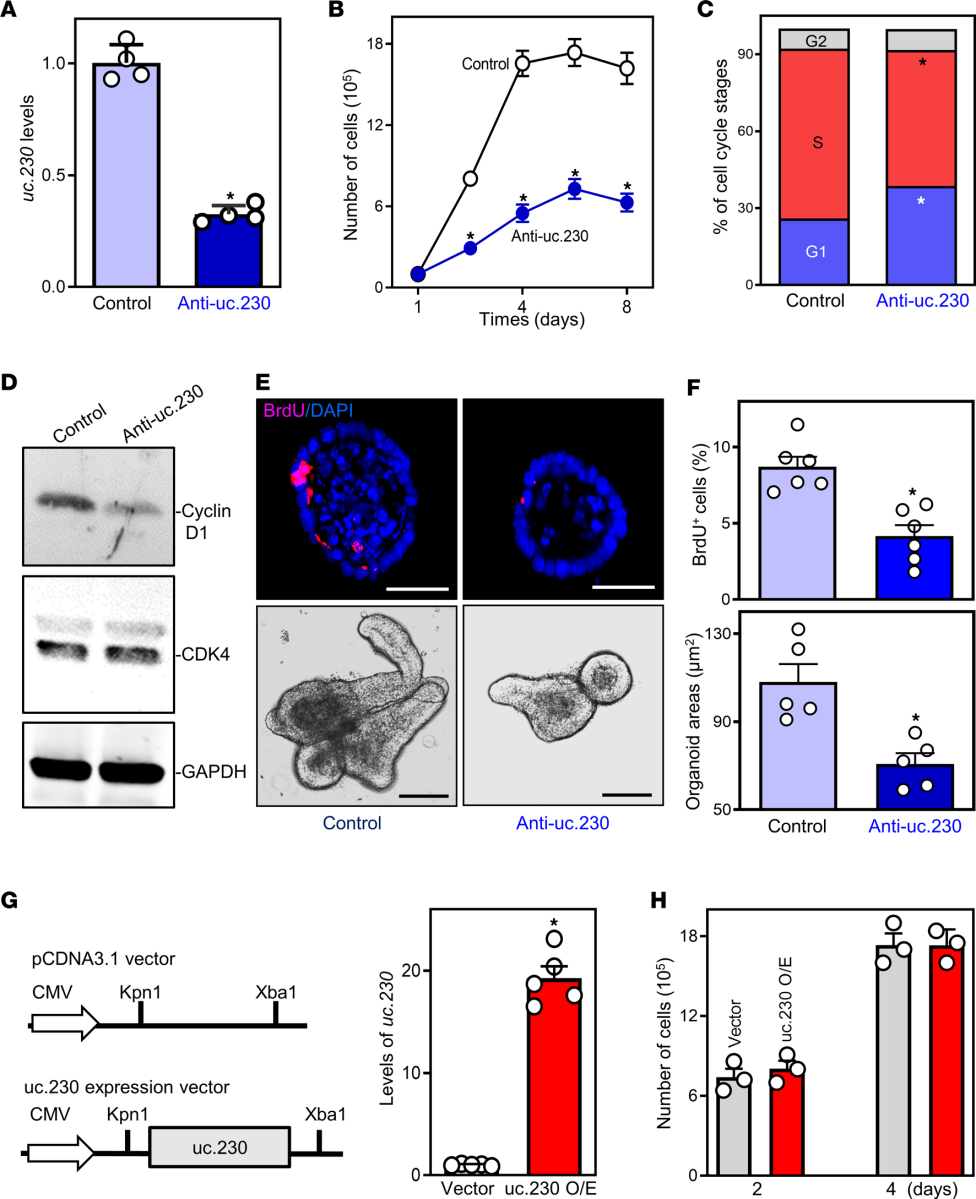
*uc.230* is required for IEC renewal. (**A**) Levels of *uc.230* in Caco-2 cells 48 hours after transfection with anti-uc.230 or control oligo (control) were measured by qPCR. Values are the means ± SEM (*n* = 4). *P < 0.05 compared with control. (**B**) Cell growth after *uc.230* silencing in vitro. Equal numbers of cells were seeded in 24-well plates 24 hours after transfection. *P < 0.05 compared with control (*n* = 3). (**C**) Relative G1, S, and G2/M compartments of the cell cycle were calculated from flow cytometric analysis on day 4 after anti-uc.230 transfection. *P < 0.05 compared with control (*n* = 3). (**D**) Expression levels of cyclin D1 and CDK4 in cells described in **C**. (**E**) Growth of enteroids after *uc.230* silencing ex vivo. Top, confocal analysis of BrdU (red) and DAPI (blue) on day 4 after culture; and bottom, bright field microscopy analysis of enteroids sizes. Scale bars: 100 μm. (**F**) Quantification of BrdU (*n* = 6) and surface area (*n* = 5) of enteroids described in **E**. *P < 0.05 compared with control. (**G**) Levels of *uc.230* in cells transfected with the *uc.230* expression vector (right) for 48 hours. *P < 0.05 compared with cells control vector (*n* = 5). (**H**) Cell growth on days 2 and 4 after *uc.230* overexpression (O/E) (*n* = 3). In **A**, **C**, **F**, **G**, and **H**, statistical significance was analyzed using unpaired, 2-tailed Student’s *t* tests. In **B**, statistical comparison between time-course curves was by 2-way ANOVA with Bonferroni post hoc test.

To examine the function of *uc.230* in regulating intestinal mucosal growth, we performed 2 sets of studies, first silencing and then increasing the levels of *uc.230*. First, we examined if silencing *uc.230* by using locked nucleic acid–modified (LNA-modified) oligomers inhibited the proliferation of Caco-2 cells and primarily cultured organoids derived from the small intestines of WT mice. As shown in Figure 2A, the levels of cellular *uc.230* decreased dramatically 48 hours after transfection with LNA-modified anti-uc.230 oligonucleotides (oligo) (anti-uc.230). This decrease in *uc.230* levels inhibited Caco-2 cell proliferation as demonstrated by a significant decrease in cell number (Figure 2B). Cell cycle analysis revealed that the population of S-phase cells was reduced in *uc.230*-silenced cells, along with an increase in G1-phase cells (Figure 2C). Moreover, *uc.230* silencing decreased the levels of cellular cyclin D1, although it did not alter abundance of cyclin dependent kinase 4 (CDK4) (Figure 2D). To exclude off-target effects, another anti-uc.230 oligo, anti-uc.230_-_2, was also tested and showed similar inhibitory effects on Caco-2 cell proliferation. We also examined the effect of *uc.230* silencing on proliferation of other IECs and found that *uc.230* silencing by transfection with anti-uc.230 inhibited HCT116 and IEC-6 cell proliferation. As measured by Trypan blue exclusion, transfection with anti-uc.230 did not affect the viability of Caco-2, HCT116, or IEC-6 cells.

In an ex vivo model, decreasing *uc.230* levels by transfection with anti-uc.230 also inhibited the growth of intestinal organoids. Using methods described previously (6), intestinal organoids were initiated from proliferating crypts. By 4 days after initial culture, the structures of both control and anti-uc.230–transfected intestinal organoids consisted of multiple buds and cells. However, decreasing the levels of *uc.230* by transfecting organoids with anti-uc.230 remarkably inhibited DNA synthesis in multiple cells, as shown by reduced BrdU incorporation (Figure 2, E and F), and decreased intestinal organoid size as demonstrated by a large reduction in their surface area (Figure 2, E and F).

Second, we examined the effect of increasing the levels of *uc.230* on the proliferation of Caco-2 cells. The levels of *uc.230* were increased by transfection of a plasmid vector expressing *uc.230* under control of the cytomegalovirus promoter (pCMV) promoter (Figure 2G, schematic); 48 hours later, cellular *uc.230* levels were increased robustly (Figure 2G) and specifically (levels of other T-UCRs such as *uc.447* and *uc.283* were not altered). Unexpectedly, ectopic overexpression of *uc.230* in Caco-2 cells did not alter basal cell proliferation; at 2 and 4 days after transfection, there were no significant differences in cell numbers when comparing cells transfected with the *uc.230* plasmid with cells transfected with control vector (Figure 2H). Ectopically expressed *uc.230* also failed to increase the growth of intestinal organoids. These results indicate that basal levels of *uc.230* are required for normal intestinal epithelial renewal but further increasing *uc.230* levels does not stimulate mucosal growth.

### uc.230 protects IECs against apoptosis.

**Figure 3:**
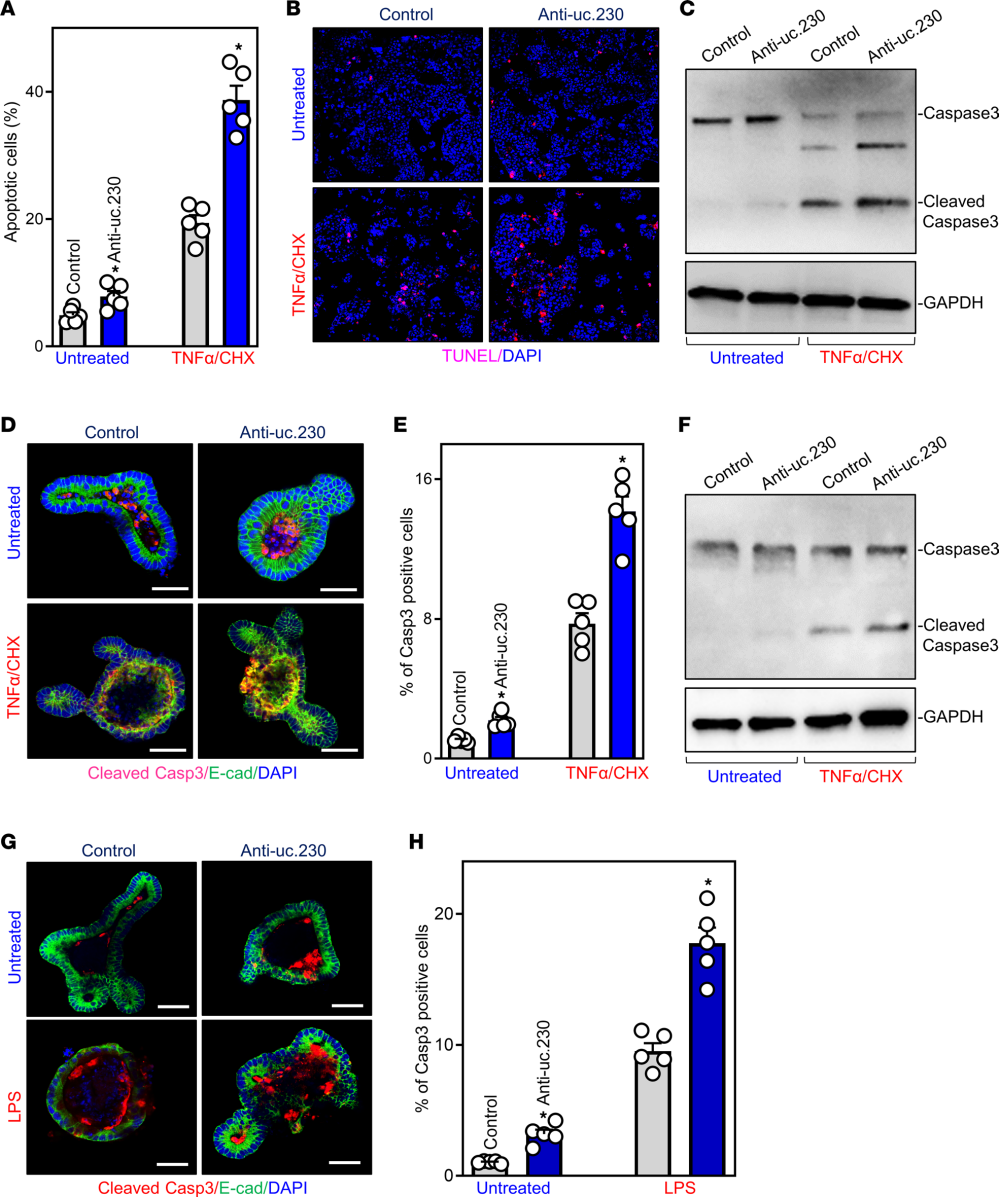
*uc.230* silencing increases IEC apoptosis. (**A**) Percentages of apoptotic cells after *uc.230* silencing. Caco-2 cells were transfected with the anti-uc.230 or control oligo (control); 48 hours after transfection, apoptosis was measured following 4 hours of treatment with TNFα (20 ng/mL) plus CHX (10 μg/mL). Values are means ± SEM (*n* = 4 or 5). *P < 0.05 compared with controls. (**B**) Images of TUNEL staining in cells described in **A**. Experiments were performed 3 separate times and showed similar results. Original magnification, ×200. (**C**) Changes in levels of caspase-3 in cells described in **A**. Whole-cell lysates were harvested, and the levels of total and cleaved procaspase-3 were assessed by Western blot analysis. GAPDH immunoblotting was performed as an internal control for equal loading. (**D**) Cleaved caspase-3 staining in enteroids after *uc.230* silencing ex vivo. Enteroids were derived from the small intestines of WT mice and transfected with anti-uc.230 or control oligo (control). Enteroids were exposed to TNFα (10 ng/mL) plus CHX (5 μg/mL) 48 hours after transfection, and apoptotic cell death was examined 3 hours thereafter. Scale bars: 100 μm. (**E**) Quantitative data of cleaved caspase-3^+^ cells in enteroids described in **D**. Values are means ± SEM (*n* = 4 or 5). *P < 0.05 compared with controls. (**F**) Changes in levels of cleaved caspase-3 in enteroids described in **D**. (**G** and **H**) Apoptotic cell death in enteroids exposed to LPS after *uc.230* silencing. Enteroids were treated with LPS (50 μg/mL) and changes in the levels of cleaved caspase-3 were examined 24 hours thereafter. *P < 0.05 compared with controls (*n* = 5). Scale bars:100 µm. In **A**, **E**, and **H**, statistical significance was analyzed using unpaired, 2-tailed Student’s *t* tests.

To investigate whether *uc.230* regulates apoptosis in the intestinal epithelium, we first asked if decreasing endogenous levels of *uc.230* in vitro and ex vivo would induce apoptosis. As shown in Figure 3A, compared with control transfection, *uc.230* silencing with anti-uc.230 transfection alone significantly increased the percentage of apoptotic Caco-2 cells. Moreover, silencing *uc.230* strikingly enhanced TNFα/CHX-induced Caco-2 cell apoptosis (Fig. 3A). When Caco-2 cells were exposed to TNFα/CHX for 4 hours, morphological features characteristic of programmed cell death, confirmed by increased levels of TUNEL staining (Figure 3B) and cleaved caspase-3 (Figure 3C), were observed in both control and *uc-230*-deficient cells. Nonetheless, the number of apoptotic cells in uc.230-silenced cells was significantly greater than that observed for cells transfected with Con-oligo after administration of TNFα/CHX (Figure 3B).

We also found that reducing *uc.230* levels by transfection with anti-uc.230 in an ex vivo model, intestinal organoids generated from WT mice, enhanced both spontaneous and TNFα/CHX-induced apoptosis. As shown in Figure 3D, spontaneous apoptosis in primary intestinal organoids cultures, marked by cells staining for cleaved caspase 3, was detected only at the luminal regions; the intensity of luminal caspase-3 staining increased significantly in anti-uc.230–transfected organoids compared with organoids transfected with Con-oligo (Figure 3E). After exposure to TNFα/CHX for 4 hours, apoptotic cells, detected by a dramatic increase in cleaved caspase-3 staining, were observed in both control and *uc.230*-silenced organoids (Figure 3E). However, compared with Con-oligo–transfected control organoids, TNFα/CHX-treated *uc.230*-deficient organoids demonstrated a significant increase in the intensity of caspase-3 staining (Figure 3E). The assessments of TNFα/CHX-induced apoptosis in intestinal organoids were confirmed by increased levels of cleaved caspase-3 protein in both groups as measured by Western blotting analysis, with augmented caspase-3 activation in *uc.230*-deficient organoids (Figure 3F). Furthermore, silencing *uc.230* also enhanced LPS-induced cell death in organoids, as evidenced by a significant increase in the number of caspase-3^+^ cells in *uc.230*-deficient organoids after exposure to LPS, compared with controls (Figure 3, G and H).

**Figure 4:**
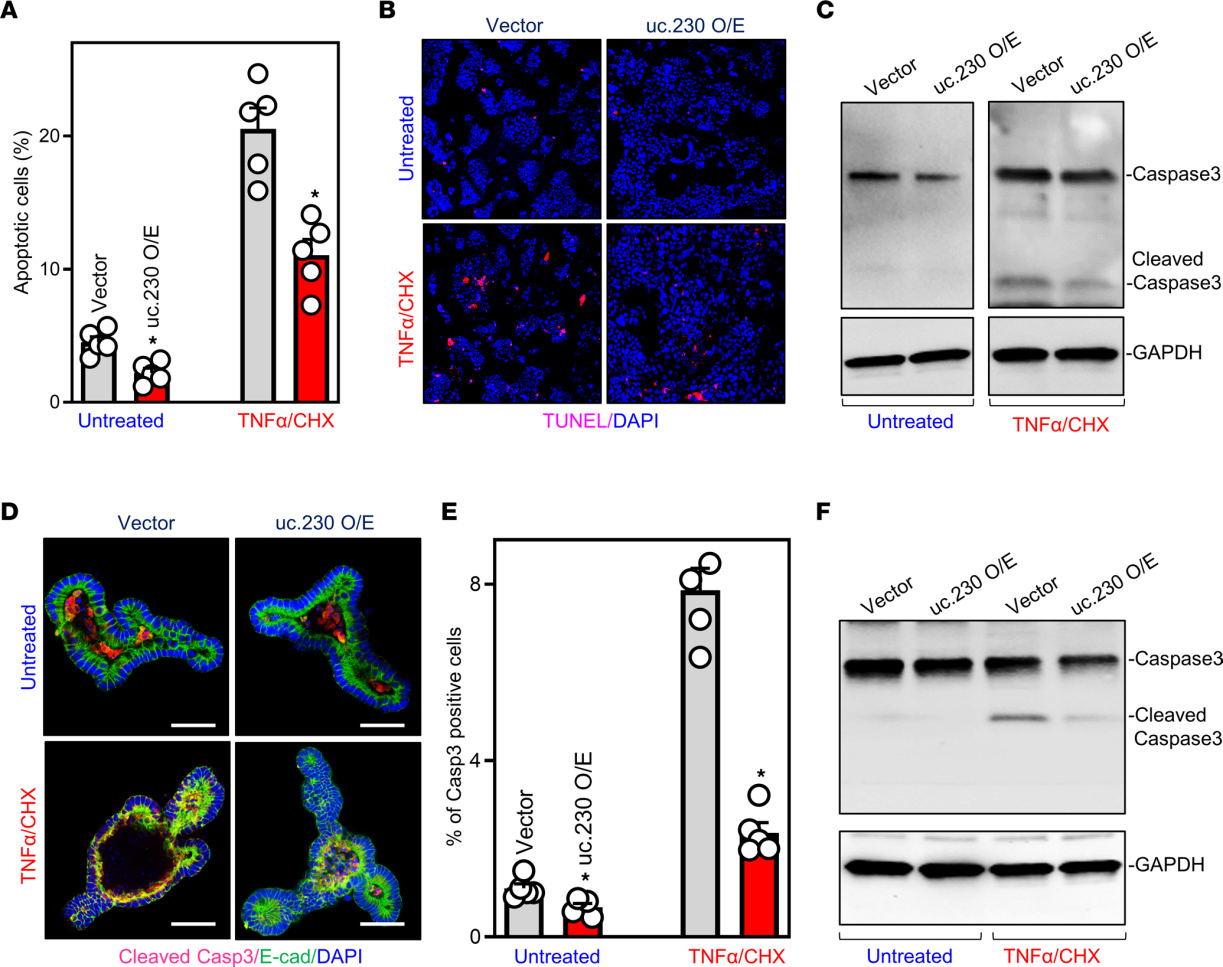
Ectopically overexpressed *uc.230* protects IECs against apoptosis. (**A**) Percentages of apoptotic cells after *uc.230* overexpression. Cells were treated with TNFα/CHX 48 hours after transfection with *uc.230* expression vector or control emptying vector, and apoptosis was measured 4 hours thereafter. Values are means ± SEM (*n* = 5). *P < 0.05 compared with control vector. (**B**) Images of TUNEL staining in cells described in **A**. Experiments were repeated 3 separate times and showed similar results. Original magnification, ×200. (**C**) Levels of cleaved caspase-3 in cells described in **A**. (**D**) Cleaved caspase-3 staining in intestinal organoids after *uc.230* overexpression. Organoids were exposed to TNFα/CHX 48 hours after transfection with *uc.230* expression vector or control vector, and apoptotic cell death was examined 3 hours thereafter. Scale bars: 100 μm. (**E**) Quantitative data of cleaved caspase-3^+^ cells in organoids described in **D**. Values are means ± SEM (*n* = 5). *P < 0.05 compared with control vector. (**F**) Changes in levels of cleaved caspase-3 in intestinal organoids treated as described in **D**. In **A** and **E**, statistical significance was analyzed using unpaired, 2-tailed Student’s *t* tests.

Next, we asked if *uc.230* overexpression impacted IEC apoptosis. Notably, in both Caco-2 cells and intestinal organoids (enteroids), increasing the levels of *uc.230* elicited striking anti-apoptotic effects (Figure 4). Ectopically expressing *uc.230* by transfection with a *uc.230* expression vector reduced both spontaneous and TNF-α/CHX-induced Caco-2 cell apoptosis. The percentages of apoptotic cells (Figure 4A), TUNEL staining (Figure 4B), and levels of cleaved caspase-3 protein (Figure 4C) in cells overexpressing *uc.230* were lower than those seen in cells transfected with control vector after exposure to TNFα/CHX. In an ex vivo model, ectopic overexpression of *uc.230* also protected intestinal enteroids against TNFα/CHX-induced apoptosis, as demonstrated by a significant decrease in the percentages of caspase-3^+^ cells (Figure 4, D and E) and the levels of cleaved caspase-3 (Figure 4F) in *uc.230*-transfected enteroids compared with organoids transfected with control vector.

**Figure 5:**
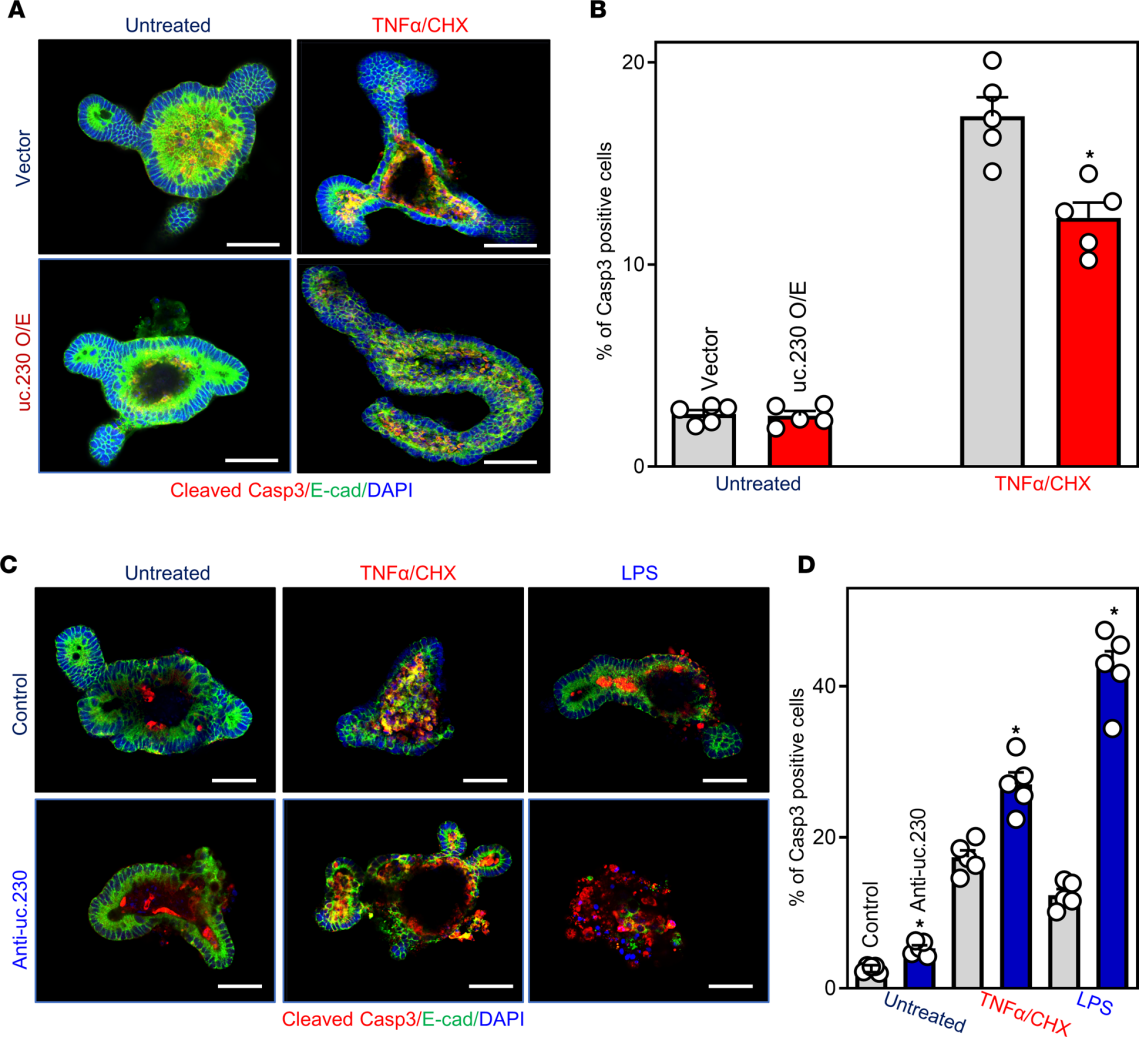
In the absence of endogenous HuR, *uc.230* protects enteroids from apoptosis. (**A**) Cleaved caspase-3 staining in HuR-deficient enteroids after *uc.230* overexpression. Enteroids were derived from the small intestine of IE-HuR^–/–^ mice and transfected with *uc.230* expression or control vector. Enteroids were exposed to TNFα/CHX 48 hours after transfection, and apoptotic cell death was examined 3 hours thereafter. Scale bars: 100 μm. (**B**) Quantitative data of cleaved caspase-3^+^ cells in organoids described in **A**. Values are means ± SEM (*n* = 5). *P < 0.05 compared with control vector. (**C** and **D**) Apoptotic cell death in enteroids after *uc.230* silencing. HuR-deficient enteroids were treated with either TNFα/CHX or LPS 48 hours after transfection with anti-uc.230 or control oligo (control). Changes in cleaved caspase-3 staining were examined after 3 hours for TNFα/CHX–treated organoids and 24 hours for LPS-treated enteroids. *P < 0.05 compared with control (*n* = 5). Scale bars: 100 μm. In **B** and **D**, statistical significance was analyzed using unpaired, 2-tailed Student’s *t* tests.

HuR, a master posttranscriptional regulator of intestinal epithelial homeostasis associated with many aspects of intestinal mucosal pathology (31, 32), is commonly dysfunctional in IBD (6). To expand the potential clinical relevance of our current findings, we explored whether *uc.230* protects the epithelium against apoptosis in HuR-deficient enteroids. To do so, intestinal organoids were derived from tissues with intestinal epithelial-selective HuR deletion (IE-*HuR*^–/–^) mice (31). Consistent with previous reports (6, 33), compared with enteroids generated from control littermates or WT mice, HuR-deficient enteroids exhibited reduced growth and increased susceptibility to TNF-α/CHX-induced apoptosis. However, in the absence of endogenous HuR, increasing *uc.230* levels by transfection with a *uc.230* expression vector protected enteroids against TNFα/CHX-induced apoptosis (Figure 5, A and B). After TNFα/CHX treatment, compared with enteroids transfected with control vector, there were significantly fewer caspase-3^+^ cells in HuR-deficient enteroids overexpressing *uc.230*. In contrast, silencing *uc.230* in HuR-deficient enteroids augmented both spontaneous and TNFα/CHX-induced apoptosis (Figure 5, C and D). The number of caspase-3^+^ cells increased in uc.230-silenced enteroids compared with organoids transfected with Con-oligo (Figure 5, C and D). After LPS treatment, some enteroids with combined HuR and *uc.230* deficiency exhibited massive cell death (> 70% of caspase-3^+^ cells). Together, these results provide strong evidence that *uc.230* is an intestinal epithelial anti-apoptotic factor whose deficiency predisposes IECs to apoptosis.

### uc.230 is required for maintaining epithelial barrier function.

**Figure 6:**
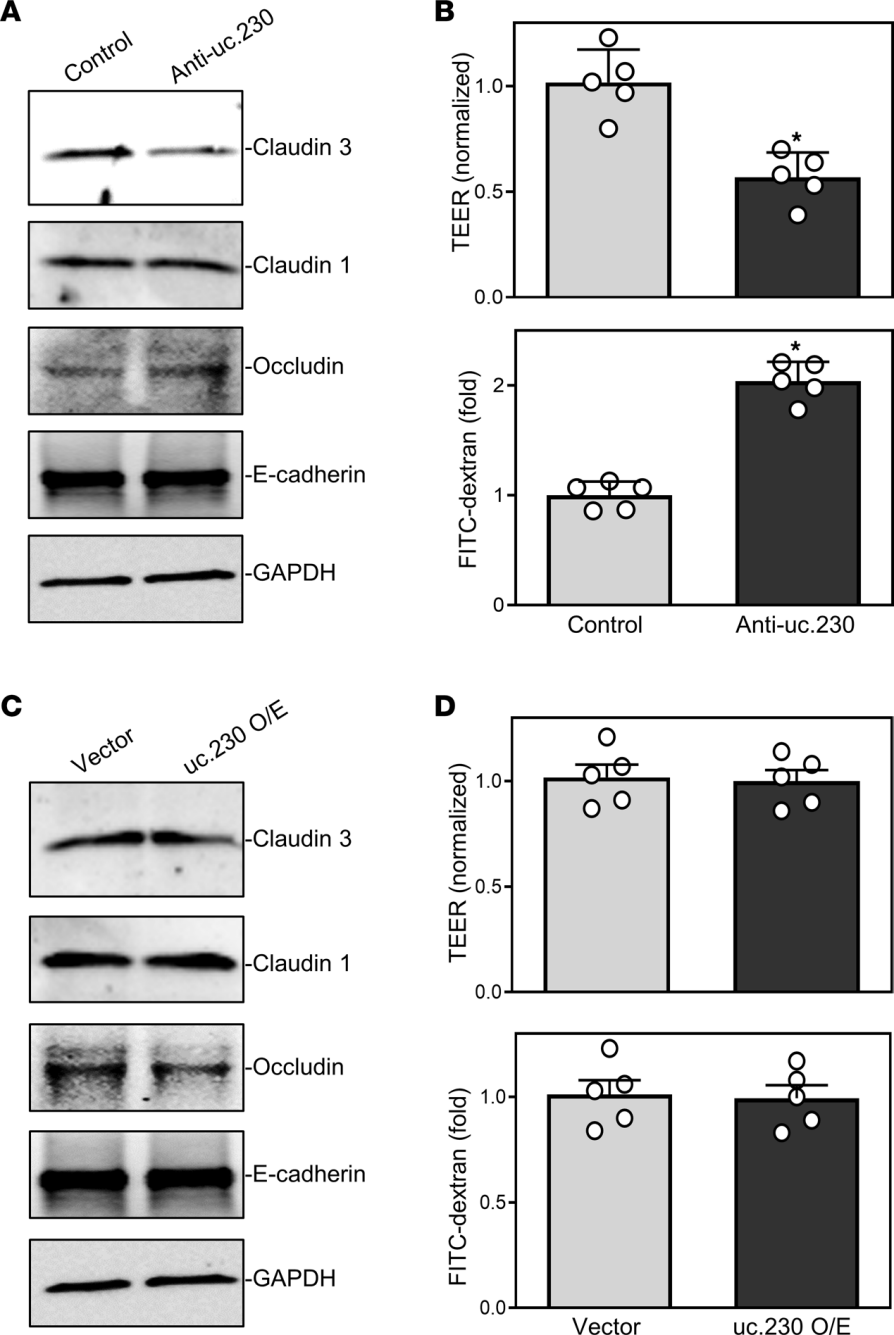
*uc.230* is essential for intestinal epithelial barrier function in vitro. (**A**) Representative immunoblots of tight and AJ proteins after *uc.230* silencing as examined by Western blotting. Caco-2 cells were transfected with anti-uc.230 or control oligo (control), and whole cell lysates were harvested 48 hours thereafter. Equal loading was monitored by assessing GAPDH levels. Experiments were performed 3 separate times and showed similar results. (**B**) Changes in epithelial barrier function as indicated by changes in TEER (top) and FITC-dextran paracellular permeability (bottom) in cells described in **A**. TEER assays were performed using 12 mm Transwell filters; and paracellular permeability was assayed by adding the membrane-impermeable trace molecule FITC-dextran to the insert medium. Values are the means ± SEM (*n* = 5). *P < 0.05 compared with control. (**C** and **D**) Effect of ectopically overexpressed (O/E) *uc.230* on epithelial barrier function. The levels of various junctional proteins in **C** and epithelial barrier function in **D** were examined (*n* = 5) 48 hours after transfection with *uc.230* expression vector or control vector. In **B** and **D**, statistical significance was analyzed using unpaired, 2-tailed Student’s *t* tests.

To elucidate the role of *uc.230*, if any, in regulating intestinal epithelial barrier function, we examined the effects of *uc.230* silencing on the expression of TJ and adherens junction (AJ) proteins in Caco-2 cells. Reducing *uc.230* levels inhibited expression of the TJ protein CLDN3 but failed to alter cellular levels of other TJ proteins, such as CLDN1 and occludin, or an AJ protein, E-cadherin (Figure 6A). In uc.230-silenced cells, CLDN3 protein levels were approximately 60% lower than observed in control cells transfected with Con-oligo. Importantly, *uc.230* silencing disrupted epithelial barrier function in an in vitro model, as evidenced by reduced transepithelial electrical resistance (TEER) (Figure 6B) and increased paracellular flux of FITC-dextran (Figure 6B). As we observed with intestinal epithelial renewal, ectopically expressed *uc.230* did not alter CLDN3 expression (Figure 6C) or epithelial barrier function (Figure 6D). The levels of CLDN3, TEER, and paracellular permeability to FITC-dextran in cells overexpressing *uc.230* were indistinguishable from those observed in cells transfected with control vector. Although induced apoptosis and inhibition of IEC proliferation also contribute to the barrier dysfunction observed in uc.230-deficient cells, these data strongly suggest that basal levels of endogenous *uc.230* are crucial to maintaining normal CLDN3 expression and epithelial barrier function.

### Inhibition of uc.230 enhances DSS-induced colonic mucosal injury and delays repair.

**Figure 7:**
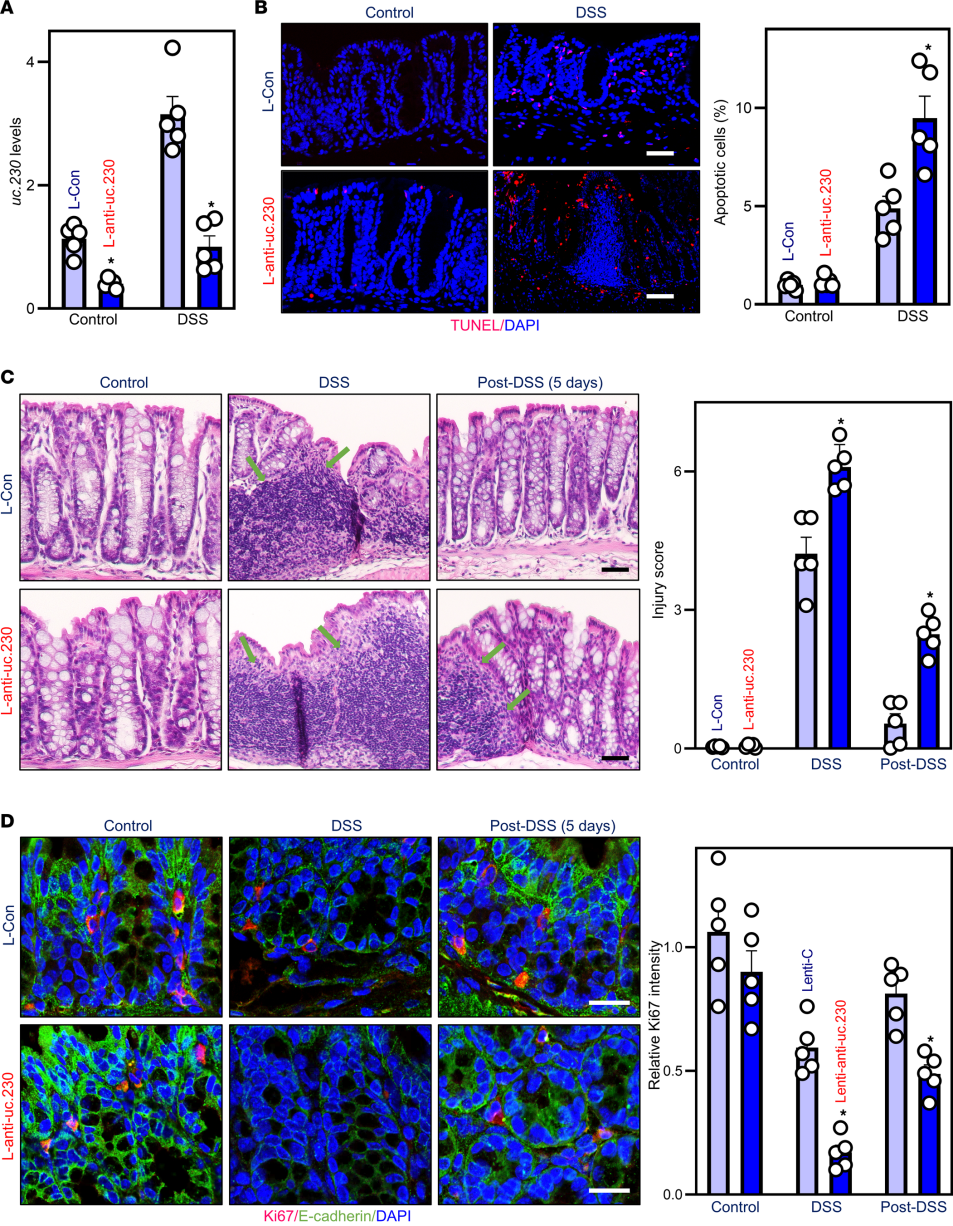
In mice, inhibition of *uc.230* enhances colonic mucosal injury and delays repair of damaged mucosa. (**A**) Levels of mucosal *uc.230* in the colons on day 5 after infection with L–anti-uc.230 or L-Con in mice treated with or without DSS. Values are the means ± SEM (*n* = 5). *P < 0.05 compared with mice infected with L-Con. (**B**) Apoptotic cells in the colonic mucosa of mice described in **A**, as measured by TUNEL staining. Scale bar: 50 μm. Values are means ± SEM (*n* = 5). *P < 0.05 compared with L-Con group. (**C**) Micrographs of the colonic mucosa in L-Con– or L–anti-uc.230–infected mice treated with water (sham; left), 3% DSS for 5 days (DSS) (middle), and 3% DSS for 5 days, followed by 5-day water feeding (Post-DSS; right). Arrows indicate mucosal inflammation. Values are the means ± SEM (*n* = 5). *P < 0.05 compared with L-Con. (**D**) Ki67 staining of the colonic mucosa in mice described in **C**. *P < 0.05 compared with L-Con. In **A**–**D**, statistical significance was analyzed using unpaired, 2-tailed Student’s *t* tests.

To examine the in vivo importance of *uc.230* in regulating intestinal mucosal injury, we examined the effect of reducing *uc.230* levels on DSS-induced colonic mucosal injury in mice. A recombinant anti-uc.230 lentiviral expression vector (L–anti-uc.230) was generated to antagonize *uc.230* in mice as described previously (3, 13). Infecting mice with L–anti-uc.230 for 10 days did not cause diarrhea, weight loss, hair loss, or reduced activity. Compared with animals infected with the control lentiviral vector (L-Con), *uc.230* levels were reduced by approximately 65% in mice infected with L–anti-uc.230 for 5 days (Figure 7A). Infection with L–anti-uc.230 also prevented DSS-induced increases in colon mucosal *uc.230* levels (Figure 7A). Importantly, reducing *uc.230* levels augmented DSS-induced apoptosis in the colonic mucosa (Figure 7B), as examined by TUNEL staining (33, 34). Exposure to DSS for 5 days induced apoptosis in both mice infected with L-Con or L–anti-uc.230, but *uc.230*-antagonized mice exhibited a significant increase in the rate of apoptotic cell death compared with mice infected with L-Con. Reducing *uc.230* levels by infection with L–anti-uc.230 consistently enhanced DSS-induced colonic mucosal injury and inhibited repair of the damaged mucosa (Figure 7C). In *uc.230*-deficient mice compared with those infected with L-Con, inflammatory injury/erosions increased significantly on day 5 after DSS treatment. In L-Con–infected mice, the mucosa was rapidly repaired following resumption of normal drinking water, and mucosal histology was nearly normal from day 5 onward. In contrast, substantial mucosal infiltration with granulocytes and erosions persisted in *uc.230*-antagonized mice despite switching to unadulterated water (Figure 7C). Moreover, following DSS-induced colonic mucosal injury in mice infected with L–anti-uc.230, the reduced levels of *uc.230* inhibited colonic mucosal renewal, as evidenced by significantly fewer Ki67^+^ cells in the mucosa of *uc.230*-deficient mice compared with L-Con mice (Figure 7D). The levels of E-cadherin immunostaining also decreased in both mice infected with L-Con or L–anti-uc.230 after exposure to DSS for 5 days but reducing *uc.230* by infection with L–anti-uc.230 had no additional effect on this reduction. These results indicate that, because it impairs epithelial defense and inhibits mucosal regeneration, reducing *uc.230* expression both enhances DSS-induced mucosal injury and delays its repair.

### uc.230 increases CUGBP1 via interaction with miR-503.

**Figure 8:**
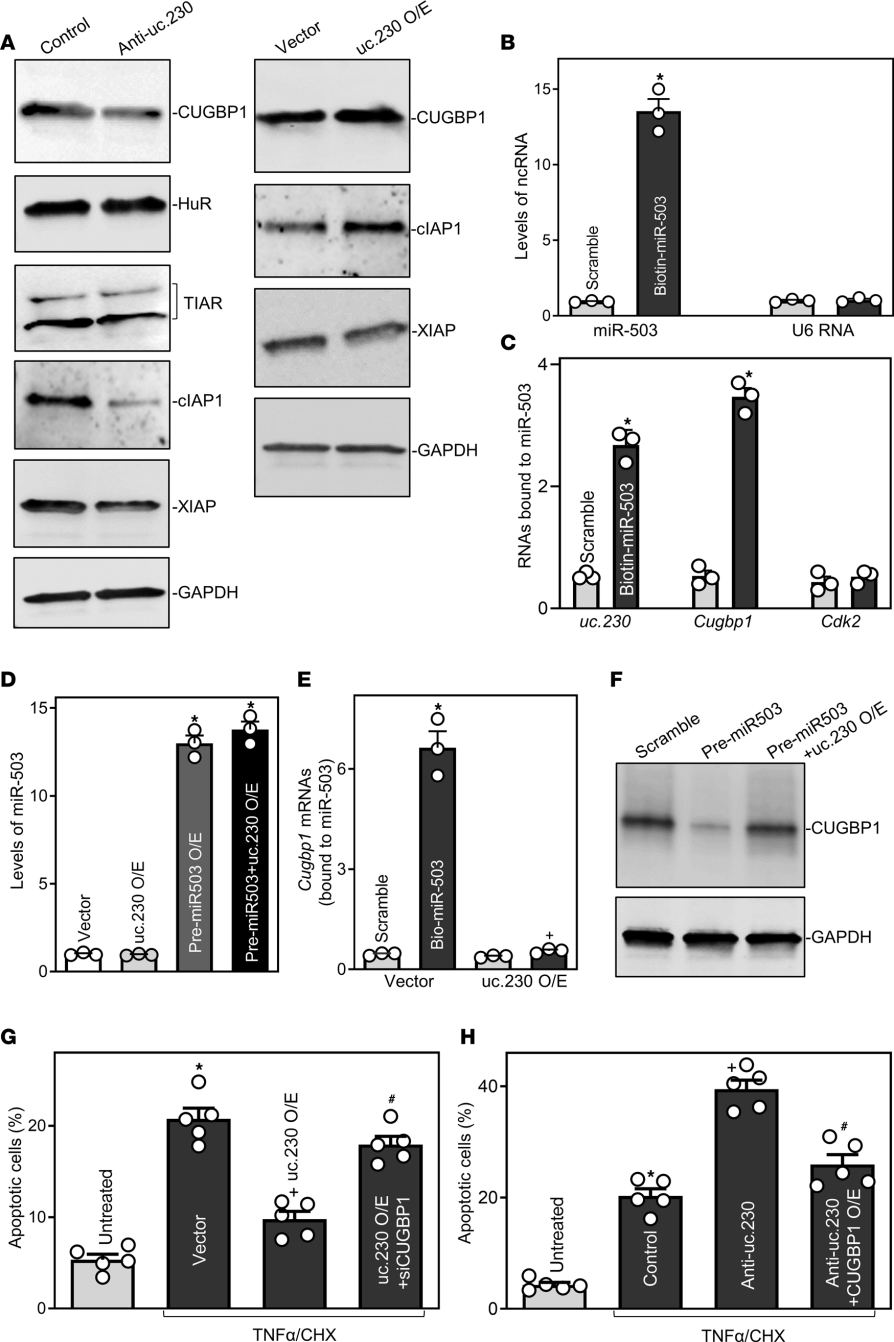
*Uc.230* prevents apoptosis by increasing CUGBP1 via interaction with miR-503. (**A**) Immunoblots of RBPs and anti-apoptotic proteins after silencing or overexpressing *uc.230*. Caco-2 cells were transfected with anti-uc.230 or uc.230 expression vector, and levels of various proteins were examined 48 hours after the transfections. (**B**) Levels of biotinylated miR-miR-503 (left) and *U6* RNA (right) 24 hours after transfection with biotinylated miR-503. Values are the means ± SEM (*n* = 3). *P < 0.05 compared with cells transfected with scrambled oligomer. (**C**) Binding of biotinylated miR-503 to *uc.230* and mRNAs *Cugbp1* and *Cdk2* in cells described in **B**. *P < 0.05 compared with scrambled oligomer (*n* = 3). (**D**) Levels of miR-503 48 hours after cells were transfected with pre–miR-503 alone or cotransfected with pre–miR-503 and *uc.230* expression vector. ^*^P < 0.05 compared with cells transfected with control vector or uc.230 expression vector alone (*n* = 3). (**E**) Effect of increasing *uc.230* on miR-503/*Cugbp1* mRNA association. Cells were transfected with bio-miR-503 or scrambled oligomer 24 hours after cells were transfected with *uc.230* expression vector. The levels of *Cugbp1* mRNA in the materials pulled down by miR-503 were examined 24 hours thereafter. *P < 0.05 compared with cells transfected with scrambled oligomer and ^+^*P* < 0.05 compared with cells transfected with bio-miR-503 with control vector. (**F**) Representative immunoblots of CUGBP1 48 hours after cells were transfected with pre–miR-503 alone or cotransfected with pre–miR-503 and *uc.230* expression vector. Experiments were repeated 3 separate times and showed similar results. (**G** and **H**) Percentages of TNFα/CHX–induced apoptosis in cells cotransfected with *uc.230* expression vector and siCUGBP1 or cotransfected with anti-uc.230 and CUGBP1 expression vector. Cells were treated with TNFα/CHX 48 hours after cotransfection, and apoptosis was measured 4 hours thereafter. Values are means ± SEM (*n* = 5). *P < 0.05 compared with untreated group; ^+^*P* < 0.05 compared with cells transfected with vector or control oligo; and ^#^*P* < 0.05 compared with cells transfected with uc.230 expression vector or anti-uc.230 alone. In **B**, **C**, and **E**, statistical significance was analyzed using unpaired, 2-tailed Student’s *t* tests, while comparisons of means between more than 2 groups were performed by 1-way ANOVA with Tukey’s multiple-comparison test for **D**, **G**, and **H**.

To investigate the molecular mechanisms underlying the actions of *uc.230* in maintaining intestinal epithelial homeostasis, we tested the possibility that *uc.230* regulates apoptosis by altering expression of RBPs. We carried out 3 sets of experiments in cultured Caco-2 cells. First, we determined the effect of *uc.230* on expression of gut mucosal enriched RBPs and demonstrated that silencing *uc.230* specifically decreased the levels of CUGBP1 but did not alter the cellular abundance of other RBPs, i.e., HuR and TIAR (Figure 8A). Interestingly, CUGBP1 influences many aspects of the cellular response to apoptotic signals (26, 34), and our results show that reduced CUGBP1 in *uc.230*-silenced cells is associated with decreased levels of 2 inhibitors of apoptosis (IAP): c-IAP1 and XIAP. Inversely, overexpressing *uc.230* increased the abundance of CUGBP1, but did not alter c-IAP and XIAP levels (Figure 8A).

Next, we determined if *uc.230* stimulated CUGBP1 expression by decreasing the association of miR-503 with *Cugbp1* mRNA. As miR-503 constitutively represses CUGBP1 translation by binding to *Cugbp1* mRNA (34), we examined the association of *uc.230* with miR-503 using RNA pulldown assays with biotin–miR-503 (28). As shown in Figure 8B, cells transfected with biotin-labeled miR-503 exhibited increased miR-503 levels but displayed no changes in U6 RNA. We found that levels of both *uc.230* and *Cugbp1* mRNA were highly enriched in materials pulled down from cells transfected with biotin-labeled miR-503 but not from cells transfected with scrambled control miRNA (Figure 8C). The interactions of miR-503 with *uc.230* and *Cugbp1* mRNA are specific; increasing the levels of biotin-labeled miR-503 did not increase its binding to *Cdk2* mRNA.

As shown in Figure 8D, increasing the levels of *uc.230* did not alter miR-503 levels in cells cotransfected with precursor–miR-503 (pre–miR-503) and *uc.230* expression vector, but blocked binding of miR-503 to *Cugbp1* mRNA (Figure 8E). There were no significant differences in the levels of *Cugbp1* mRNA in the pulldown materials between cells transfected with biotin-labeled miR-503 and cells transfected with biotin-labeled control scrambled oligomer after *uc.230* overexpression (Figure 8E). Like previous observations (17, 34), cotransfection with biotin-labeled miR-503 and *uc.230* expression vector did not alter the total input of *Cugbp1* mRNA. Consistent with these results, in cells overexpressing miR-503, elevation of *uc.230* prevented the inhibition of CUGBP1 expression (Figure 8F). Levels of CUGBP1 in cells cotransfected with pre–miR-503 and the *uc.230* expression vector were identical to those observed in cells transfected with control scrambled oligomer.

Then, we tested if altered CUGBP1 expression played a role in *uc.230*-regulated apoptosis. As shown in Figure 8G, in cells overexpressing *us.230*, decreasing the levels of CUGBP1 by transfection with CUGBP1-directed siRNA (siCUGBP1) attenuated *uc.230*-mediated protection against TNFα/CHX-induced apoptosis, whereas ectopically expressing CUGBP1 in uc.230-silenced cells attenuated the increase in apoptosis following exposure to TNFα/CHX (Figure 8H). Because CUGBP1 is a potent and biological anti-apoptotic factor in the intestinal epithelium and its cellular abundance is tightly regulated (9, 26, 34), these findings strongly support the conclusion that *uc.230* protects IECs against apoptosis at least in part by serving as a natural sponge for miR-503, and thereby preserving CUGBP1 expression.

## Discussion

The striking evolutionary retention of UCRs in mammalian genomes suggests that T-UCRs have important functions in gut physiology and disease. In this regard, *uc.173* promotes intestinal mucosal growth and enhances epithelial barrier function, and intestinal mucosal *uc.173* levels are reduced in CD (16, 17). In the present study, we observed that *uc.230* is highly expressed in the intestinal epithelium and its cellular abundance changes remarkably in mucosal injury and disease. Our work demonstrates that *uc.230* sustains intestinal epithelial homeostasis, as it is required for IEC proliferation and epithelial barrier function and protects the epithelium against apoptosis. Reducing tissue levels of *uc.230* in mice enhances DSS-induced mucosal injury and delays restitution with normal mucosa. Molecular characterization revealed that *uc.230* may function as a molecular decoy for miRNAs in IECs: *uc.230* increased CUGBP1 expression by inhibiting miR-503 binding to *Cugbp1* mRNA. These findings expand our knowledge of the biological functions of T-UCRs in the intestinal mucosa and represent a major conceptual advance linking T-UCR *uc.230* with epithelial renewal and defense. Given that intestinal mucosal *uc.230* expression levels are increased in UC, our results provide a strong rationale for developing therapeutics directed at the *uc.230*/CUGBP1 axis to preserve intestinal epithelial integrity in the clinical setting.

Our results indicate that *uc.230* regulates intestinal epithelial homeostasis by altering IEC proliferation, apoptosis, and TJ protein expression. Transcribed from the ultraconserved element located at the negative strand of human chromosome 7 (18), cellular *uc.230* levels are tightly regulated in the intestinal epithelium. Mucosal *uc.230* levels are substantially increased in the colon of DSS-treated mice, in the small intestine of fasted animals, and in cultured Caco-2 cells after depletion of cellular polyamines. Although *uc.230* levels were not altered in ileal mucosa obtained from patients with CD, colonic mucosal tissues from patients with UC had more abundant *uc.230* than observed in control tissues. Our studies in cultured cells and in ex vivo enteroids strongly support the view that *uc.230* is essential for normal intestinal epithelial renewal and barrier function and is involved in epithelial defense by protecting IECs against apoptosis in the presence or absence of endogenous HuR. To induce apoptosis and cell death in cultured cells and intestinal organoids, we used 2 pathological challenges, TNFα/CHX and LPS, that exemplify clinical gut mucosal inflammation and injury (1, 15, 24, 33). TNFα and LPS are common pathological factors that impair epithelial homeostasis in critical illness. In support of our findings, several T-UCRs were found to play a critical role in gut mucosal physiology and pathology (19, 35–37). Besides *uc.173* (16, 17), *uc.261* participates in the control of gut permeability and its overexpression contributes to barrier dysfunction in CD (22). Mucosal levels of *uc.291^+^A*, *uc.144^+^A*, *uc.261^+^A*, and *uc.477^+^A* were increased in the intestines of subjects with CD, whereas *uc.166^+^A*, *uc.141^+^A*, *uc478^+^*, and *uc.479^+^* abundances were reduced (22, 35, 37), although their exact roles in IBD remain unknown. Also, decreased levels of *uc.77^–^* promote colorectal cancer proliferation by inhibiting FBXW8-mediated CDK4 degradation (38), while *uc.63^+^* induces gastric cancer progression via NF-κB signaling (39).

Our present results also show that *uc.230* functions as a molecular sponge for miR-503 in IECs, thereby decreasing the availability of miR-503 for binding to *Cugbp1* mRNA and increasing CUGBP1 expression. CUGBP1 protects IECs against apoptosis by upregulating c-IAP and XIAP and is intimately involved in maintaining intestinal epithelial integrity (34, 40). Although ectopically overexpressed *uc.230* failed to alter miR-503 levels, it prevented miR-503 association with the *Cugbp1* mRNA and restored CUGBP1 expression in cells overexpressing miR-503. In keeping with our present observations, several T-UCRs were identified as miRNA sponges that reduce the activity of the target miRNAs without necessarily affecting their biogenesis (36, 41). For example, in bladder tissues, *uc.8^+^* traps miR-596 and lowers the availability of this miRNA to bind *MMP9* mRNA (42), while, in lung cancer cells, *uc.339* acts as a decoy RNA for 3 miRNAs, including miR-339-3p, miR-663-3p, and miR-95-5p (21). *Uc.173* functions as a natural decoy RNA for miR-29b and its induction prevents miR-29b–mediated inhibition of CLDN1 translation (17). On the other hand, T-UCRs also regulate miRNA activity at another layer. It was reported that *uc.283^+^A* controls miR-195 biogenesis by preventing primary miRNA cleavage to pre-miRNA through inhibition of Drosha-mediated processing (23). *Uc.173* interferes with miR-195 stability via RNA/RNA interaction between the upstream of 3’ terminus of the primary miR-195 and a specific sequence located at *uc.173* (16). Because CUGBP1 is highly expressed in the intestinal epithelium and its translation is tightly regulated by miR-503 in response to stress (34), it is likely that *uc.230* affects gene regulatory programs governing intestinal epithelial homeostasis at least partially by altering CUGBP1 expression via the *uc.230* interaction with miR-503.

The exact mechanism by which cellular levels of *uc.230* in the intestinal epithelium are regulated remains unknown. Previous evidence suggests that T-UCRs are altered at the transcriptional level and that DNA methylation and miRNA-mediated regulation are responsible for T-UCR modulation (19, 36). Like the downregulation of protein-coding genes, the loss of adequate T-UCR expression has been linked to the presence of CpG island promoter hypermethylation (36). Almost half of the T-UCR–associated CpG island in all tissues is unmethylated, while the other half shows tissue-specific T-UCR CpG island methylation (43–45). Epigenetic inactivation by CpG island hypermethylation of several T-UCRs such as *uc.160*, *uc.283^+^A*, and *uc.346^+^* occurs in a wide spectrum of human cancer cells and these changes in promoter methylation modulate the expression of T-UCRs (46). Wnt signaling also plays an important role in regulating T-UCR expression, and *uc.158^–^* is activated by the Wnt/β-catenin pathway in liver cancers and drives their growth (47). Interestingly, Wnt activity is often increased in concert with intestinal mucosal erosion, inflammation, and repair after acute injury (48, 49), like the patterns of increased *uc.230* we observed in DSS-induced colitis in mice and UC in humans. Ongoing efforts in our laboratory will determine whether *uc.230* induction in colitis results from intestinal epithelial activation of the Wnt pathway or CpG island demethylation.

The results of the present study may have important clinical implications because *uc.230* levels are increased in intestinal mucosa from patients with UC. Impaired intestinal epithelial homeostasis and protection against or recovery from damage are separate but closely related biological processes (3, 5). Changes in *uc.230* levels and other factors observed in damaged mucosal tissues of patients with UC and mice with DSS-induced colitis are likely to be highly relevant to further injury, disrupted tissue regeneration, or the recovery of normal intestinal epithelial homeostasis. Based on our studies using cultured IECs and intestinal organoids, rather than contributing to mucosal injury and inhibiting cell proliferation, we believe it is more likely that increased *uc.230* levels in patients with UC and mice with DSS-induced colonic mucosal injury protect against further epithelial damage and enhance mucosal repair. On the other hand, there were no significant differences in *uc.230* levels in ileal mucosal tissues obtained from control and CD subjects. Differential expression of *uc.230* in UC and CD could be a consequence of anatomic location or other differences in the underlying molecular pathology of these 2 disorders. This interesting observation is beyond the scope of the current work but will be investigated in future studies.

In summary, the results in the present communication indicate that by regulating IEC proliferation, apoptosis, and barrier function, *uc.230* plays an important role in intestinal epithelial homeostasis. We also found that *uc.230* protects IECs against apoptosis by protecting CUGBP1 expression from downregulation by miR-503. These findings elucidate the pathophysiological functions of T-UCRs in the intestinal epithelium and identify the *uc.230*/CUGBP1 axis as a potential therapeutic target to protect intestinal epithelial integrity in patients with critical disorders.

## Methods

### Chemicals and cell culture.

Caco-2 cells were purchased from the American Type Culture Collection and were maintained under standard culture conditions (28). The culture medium and FBS were purchased from Invitrogen and the biochemicals were from Sigma. Antibodies recognizing CUGBP1 (catalog SC-20003), HuR (catalog SC-5261), TIAR (catalog 610352), cIAP (clone D5G9), XIAP (catalog 610763), cyclin D1 (catalog SC-8396), CLDN1 (catalog 4933), CLDN3 (catalog D7A30), occludin (catalog 611091), E-cadherin (catalog 610182), caspase-3 (clone D175), and GAPDH (catalog SC-32233) were obtained from Santa Cruz Biotechnology and BD Biosciences, and the secondary antibody conjugated to HRP was from Sigma. All antibodies utilized in this study were validated for species specificity. Antibody dilutions used for Western blots of CUGBP1, HuR, TIAR, cIAP, XIAP, cyclin D1, CLDN1, CLDN3, occludin, E-cadherin, caspase-3, and GAPDH were 1:800 or 1000 (first Ab) and 1:2000 (second Ab), respectively, whereas antibody dilutions for immunostaining were 1:200 (first Ab) and 1:200 (second Ab). Relative protein levels were analyzed using the Bio-Rad Chemidoc and XRS system equipped with Image lab software (version 4.1). We also utilized “Quantity tool” to determine the band intensity volume; values were normalized using GAPDH as an internal loading control.

### Studies in murine and human tissues.

WT C57BL/6J mice (male and female, 6- to 9-week-old) were purchased from Jackson Labs, while IE-HuR^–/–^ and littermate (HuR^fl/fl^ Cre^–^) mice were generated by crossing HuR^fl/fl^ with villin-Cre mice as described in our previous studies (6, 31). All animals were housed in a pathogen-free animal facility at the Baltimore VA Medical Center. Acute colitis was induced in mice by adding 3% DSS (catalog D8906, Sigma) to the drinking water for 5 consecutive days as reported (27). The colonic mucosal tissues were harvested for various chemical and histological analyses. In the fasting model, animals were deprived of food but allowed free access to tap water for 48 hours. Then, two 4 cm segments taken from the middle of the small intestine were harvested from each animal as described previously (50, 51). The intestinal mucosal tissues, containing more than 91% IECs (3, 13), were scraped from the underlying smooth muscles with a glass slide and used for various measurements.

Human tissue samples were obtained from surplus discarded tissue from the Department of Surgery, University of Maryland Medical Center and a commercial tissue bank (ProteoGenex). All ileal and colonic mucosal tissue samples were divided into 2 portions: 1 for extraction of RNA and the other for histological examination. The procedures for RNA extraction and histological analysis were carried out according to the method described (50, 51).

### Enteroid culture.

Isolation and culture of primary enterocytes were conducted following the method described previously (6, 52). Briefly, primary crypts were isolated from the small intestinal mucosa harvested from WT, IE-HuR^–/–^, and littermate mice; and isolated crypts were mixed with Matrigel and cultured in IntestiCult organoid growth medium. Changes in DNA synthesis and apoptotic cell death were measured by BrdU incorporation and cleaved caspase-3 staining, respectively, while the growth of enteroids was examined by measuring the surface area of organoid horizontal cross sections under phase-contrast microscopy.

### Plasmid construction.

An expression vector containing a 1.5 kb fragment flanking the human *uc.230* locus under the control of pCMV promoter (catalog PS100001) was purchased from Origene and used to increase cellular *uc.230* as described previously (13, 16). Transient transfections were performed using the Lipofectamine reagent following the manufacturer’s recommendations (Invitrogen); 48 hours after transfection using LipofectAMINE, cells were harvested for analysis.

### Isolation of nuclear and cytoplasmic RNA.

Nuclear and cytoplasmic RNA fractions from Caco-2 cells were isolated and purified with the SurePrep Nuclear/Cytoplasmic RNA Purification Kit (catalog BP280050) from Fisher Bioreagents, following the manufacturer’s instructions. Briefly, after lysis solution was added, whole cell lysates were transferred and centrifuged at 17,000*g* for 3 minutes at 4°C. The supernatant containing cytoplasmic RNA and the pellet containing nuclear RNA were saved separately and then mixed with the binding solution. After addition of ethanol, the mixtures were applied onto a spin column and centrifuged at 17,000*g* for 1 minute at 4°C, and then RNA was eluted. *Gapdh* mRNA levels were measured as an internal control for normalization. To assess for any cross-contamination between cytoplasmic and nuclear fractions, the levels of β-tubulin (a specific cytoplasmic protein marker) and lamin B (a nuclear protein marker) were examined in the 2 fractions (13).

### qPCR and digital PCR analyses.

Total RNA was isolated using the RNeasy mini kit (catalog 217004, Qiagen), and reverse transcription PCR (RT-PCR) amplification reactions were performed as described (53). The levels of *Gapdh* PCR product were examined to monitor RNA input in RT-PCR samples. qPCR analysis was conducted using 7500 Fast Real-Time PCR Systems with specific primers, probes, and software (Applied Biosystems). For miRNA studies, miR-503 levels were also quantified by qPCR using the Taqman MicroRNA assay; small nuclear RNA (snRNA) *U6* was used as an endogenous control. All primers used for qPCR analysis were purchased from Thermo Fisher Scientific.

Digital PCR analysis was performed using QuantStudio 3D Digital PCR System (Applied Biosystem) following the manufacture’s manual as described (3, 54). Briefly, PCR reaction mixture containing complement DNA was loaded onto a 20K chip v2, and a 2-step thermocycling protocol (95°C × 10 min; 40 cycles of 94°C × 30 s, 60°C × 60 s, and 98°C × 10 min) was performed in ProFlex flat PCR system. The chip was read and analyzed using QuantStudio 3D AnalysisSuite software to compute droplet concentrations (copies/ng RNA).

### Biotin-labeled RNA pulldown assays.

Biotin-labeled miR-503 was transfected and whole-cell lysates were collected 24 hours later, mixed with Streptavidin-Dynal beads, and incubated on a rotator overnight (28). After the beads were washed thoroughly, the beads-bound RNA was isolated and subjected to RT followed by qPCR analysis. Input RNA was extracted and served as control.

### Western blot analysis.

Whole-cell lysates were prepared using 2% SDS, sonicated, and centrifuged (13,800*g*) at 4°C for 15 minutes. The supernatants were boiled for 5 minutes and size-fractionated by SDS-PAGE (7.5% acrylamide). After transferring proteins onto nitrocellulose filters, the blots were incubated with primary antibodies recognizing different proteins; following incubations with secondary antibodies, immunocomplexes were developed using chemiluminescence.

### Immunofluorescence staining.

The immunofluorescence staining procedure of intestinal organoids was carried out according to the method described previously (6, 13). Slides were fixed in 3.7% formaldehyde in PBS and rehydrated. All slides were incubated with the primary antibody against cleaved caspase-3 or E-cadherin in the blocking buffer at a concentration of 1:200 or 1:300 dilution at 4°C overnight and then incubated with secondary antibody conjugated with Alexa Fluor-594 (Molecular Probes) for 2 hours at room temperature. After rinsing 3 times, the slides were incubated with 1 μM DAPI (catalog 17895, Electron Microscopy Sciences) for 10 minutes to stain cell nuclei. Finally, the slides were washed, mounted, and viewed through a Zeiss confocal microscope (model LSM710). In addition, TUNEL staining was conducted using a commercial kit for Click-iT Plus TUNEL assay, (catalog C10618, Invitrogen) and performed following the manufacturer’s recommendation. Slides were examined in a blinded fashion by coding, and decoding was performed only after examination was completed. Images were processed using Photoshop software (Adobe).

### RNA-FISH assay.

RNA-FISH was performed with the ISH optimization kit from Exiqon (catalog 90000, Vedbaek) as described (13, 16). Briefly, the mucosa was fixed with 4% fresh paraformaldehyde (PFA) overnight and embedded in paraffin for sectioning. The slides were deparaffinized and then incubated with proteinase-K. After washes with PBS, the slides were dehydrated and hybridized with 25 nM fluorescent LNA-probe for 1 hour at 60°C. The slides were washed with SSC buffers and PBS, covered with cover slides, and then processed using a Zeiss confocal microscope.

### Measurements of epithelial barrier function in vitro.

Epithelial barrier function was examined in vitro by paracellular tracer flux assays using 12-well Transwell plates as described (14, 15, 55). FITC-dextran (70 kDa, Sigma), a membrane-impermeable molecule, served as the paracellular tracer and was added to the apical bathing wells. The basal bathing well had no added tracers and contained the same flux assay medium as in the apical compartment. All flux assays were performed at 37°C, and the basal medium was collected at different times after addition of the FITC-dextran. The concentration of the FITC-dextran in the basal medium was determined using a fluorescence plate reader with an excitation wavelength at 490 nm and an emission wavelength of 530 nm. TEER was measured with an epithelial voltmeter under open circuit conditions (WPI) as described (17, 55), and the TEER of all monolayers was normalized to that of control monolayers in same experiment.

### Statistics.

All values were expressed as the means ± SEM. Sample sizes (*n*) are mentioned on each figure legend. Unpaired, 2-tailed Student’s *t* test was used when indicated with *P* < 0.05 considered significant. When assessing multiple groups, 1-way or 2-way ANOVA was utilized with Tukey’s post hoc test to analyze multiple sequential observations (56). The statistical software used was GraphPad Instat Prism 9.0. For nonparametric analysis rank comparison, the Kruskal-Wallis test was conducted.

### Study approvals.

All animal experiments were conducted in accordance with NIH guidelines and were approved by the Institutional Animal Care and Use Committee of the University of Maryland School of Medicine and the Research and Development Committee at the Baltimore VA Medical Center. The study using human tissues was approved by the University of Maryland Institutional Review Board.

## Author contributions

TXY, SK, and XL performed most experiments and summarized data. NH, LX, HKC, and JNR participated in the experiments in vivo, immunoprecipitation assays, and experiments conducted in intestinal organoids and cultured IECs. TCH and JPR participated in experiments using human tissues and data analysis. JYW designed experiments, analyzed data, prepared figures, and drafted the manuscript. All authors reviewed the final manuscript.

## Supplementary Material

Supplemental data

## Version 1 10/10/2022

Electronic publication
