# Supplementary material for: Long noncoding RNA uc.230/CUG-binding protein 1 axis sustains intestinal epithelial homeostasis and response to tissue injury
Source: JCI Insight. 2022 Oct 10;7(19):e156612. doi: 10.1172/jci.insight.156612 (PMC9675575; doi:10.1172/jci.insight.156612)

Supplementary Figure 1. uc.230 sequences and validation

Sequence of *uc.230 (238 bp)*

GAACTGGACACATATGTTGCACAATAGGTGATTCAGAGCAATATAGCCTATGCTGTTTTATGATATG
GAAAGCAATCAAATCTTATTTGCAAAAAATTCAGATCAATTGAATTTTAATGAGAAGCAGGTGCTGG
AGTGTAGCATCCACAAAGAATTCTGGGAATGTTTAAAATTAGTCCCATATTTGCCAGGAGACAGTTG
GGTACATAAACTTTTTTATTTCTTGATTCATGGGTCT

uc.230 transcripts in three different genomes

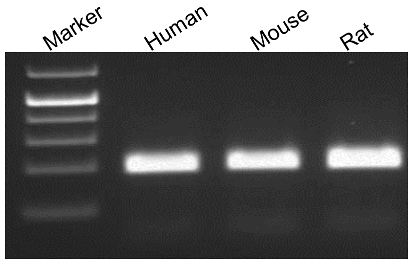

Supplement: Supplemental data [file jciinsight-7-156612-s147.pdf]
